# Synthesis, characterization, and regeneration of an inorganic–organic nanocomposite (ZnO@biomass) and its application in the capture of cationic dye

**DOI:** 10.1038/s41598-020-71261-x

**Published:** 2020-09-02

**Authors:** Kovo G. Akpomie, Jeanet Conradie

**Affiliations:** 1 Department of Chemistry, University of the Free State, Bloemfontein, South Africa; 2 Department of Pure and Industrial Chemistry, University of Nigeria, Nsukka, Nigeria

**Keywords:** Other nanotechnology, Biomaterials, Chemical engineering

## Abstract

Despite the efficiency of ZnO nanoparticle (NPs) composite adsorbents in the adsorption of various pollutants, there is presently no report on the combo of ZnONPs with biomass for adsorption. Besides, there is a dearth of information on the biosorption of celestine blue (CEB), a dye used in the nuclear and textile industry. In this study, biogenic-chemically mediated synthesis of a composite (ZnO@ACP) was prepared by the impregnation of ZnONPs onto *Ananas comosus* waste (ACP) for the adsorption of CEB. The SEM, EDX, FTIR, XRD, BET, and TGA characterizations showed the successful presence of ZnONPs on the biomass to form a nanocomposite. The uptake of CEB was enhanced by the incorporation of ZnONPs on ACP. A faster CEB adsorption onto ZnO@ACP (120 min) compared to ACP (160 min) was observed. The Langmuir (R^2^ > 0.9898) and pseudo-second-order (R^2^ > 0.9518) models were most appropriate in the description of the adsorption process. The impregnation of ZnONPs onto the biomass enhanced the spontaneity of the process and displayed endothermic characteristics. High CEB desorption of 81.3% from the dye loaded ZnO@ACP as well as efficient reusability showed the efficacy of the prepared nanocomposite for CEB adsorption.

## Introduction

The pollution of environmental water from industrial effluents contaminated with industrial by-products is on the rise. This is attributed to the rapid growth of industries, hence the increase in industrial activities across the globe. About 700,000 tons of over 100,000 commercial dyes are produced annually in industries, which makes dye the most common water pollutant^1^. Most dyes are used in the textile and paper industries and are frequently encountered in the effluents subsequently released into the environment^2,3^. These dyes present in the environmental waters can be carcinogenic, mutagenic, and result in chronic illnesses in humans and aquatic organisms^3,4^. Apart from that, they reflect or absorb light entering the water thus hindering the photosynthetic activity of aquatic plants^1^. Therefore, the removal of dyes from wastewater is necessary to abate their harmful effects on the ecosystem^5,6^. Most dye removal studies have focused on the removal of dyes such as methylene blue, congo red, malachite green, rhodamine B, methyl orange, methyl violet, reactive black, basic blue, acid yellow, brilliant green, and crystal violet^7,8^. However, research on the removal of celestine blue (CEB) is rare despite the wide use of CEB in the nuclear and textile industries. Therefore, the removal of CEB from wastewater is important. Several techniques have been harnessed for the remediation of dye-polluted water, such as filtration, coagulation, oxidation, precipitation, reduction, photocatalytic degradation, solvent extraction, and adsorption^9^. The adsorption technique is the most efficient and adsorption on bio-waste (biosorption) is promising due to the simplicity, low cost, degradability, reusability, and efficiency^10^. Hence, the use of several bio-waste for the biosorption of dyes has received much attention, but pineapple (*Ananas comosus*) peel waste (ACP) is seldom reported, despite its abundance and viable potentials^11^.

Meanwhile, recent researches have focused on the use of nanoparticles (NPs) for water treatment owing to their adsorption efficiency and potent catalytic activities^12,13^. Much attention has been given to the use of magnetic NPs and the composites for water treatment when compared to other NPs, attributed to the easy of recovery of magnetic NPs from solution by magnetic attraction^14,15^. However, the problem encountered in the recovery of other NPs from the treated water due to their small sizes has limited their application. Therefore, the impregnation of these NPs on a substrate (support) to form composites adsorbents to aid the recovery has become popular. Also, these composites adsorbents have better efficiency for water treatments due to the presence of various kinds of active sites for pollutant uptake, especially when composed of inorganic and organic sites^16^. Furthermore, among nanocomposite adsorbents, zinc oxide (ZnO) NPs composites have been reported to be highly efficient for the treatment of dye polluted water^17–23^. However, these hybrids are mainly a combo of ZnONPs with materials like activated carbon, graphene oxide, chitosan, graphene, polyaniline, and clay minerals^17,20,24–28^. Despite the desirable characteristics of biosorbents mentioned, there is presently no report on the combination of ZnONPs with biomass for water treatment. This study, therefore, reports for the first time the synthesis of a ZnONP-biomass composite (ZnO@ACP) and its application for the adsorption of CEB. This research is also significant as it provides insights into the biosorption behavior of CEB on materials, which is presently not established. The thermodynamics, kinetics, and isotherm of the biosorption process were evaluated. In addition, the mechanism of CEB uptake, as well as the regeneration and reuse of the materials, was investigated.

## Materials and methods

### Reagents and materials used

**Scheme 1:**
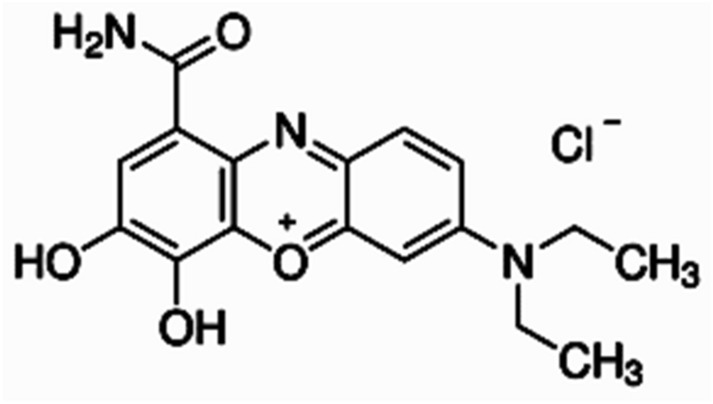
Structure of celestine blue dye.

The hydrochloric acid (HCl), nitric acid (HNO_3_), sodium hydroxide (NaOH), zinc acetate hydrate (Zn(CH_3_COO)_2_), and celestine blue (C_17_H_18_ClN_3_O_4_) were all obtained from Sigma-Aldrich. These chemicals were all analytical grade and were utilized in the experiments without any purification. The structural representation of celestine blue dye is shown in Scheme 1. The pineapple fruit (*Ananas comosus*) was obtained from Checkers mall, Mimosa, Nelson Mandela Drive, Bloemfontein, South Africa.

### Preparation of ZnO-composite adsorbent

Manual removal of the peels of *Ananas comosus* was performed using a kitchen knife. This was followed by washing with tap water to eradicate surface impurities. The peels were cut into smaller sizes to expose more surface for enhanced drying and sundried for 48 h. Thereafter, the peels were dried at 80 °C in an oven (Labcon model) for 24 h. The dried peels were pulverized into a fine powder with a laboratory pestle and mortar. To ensure the removal of water-soluble components, which could contaminate the water during the adsorption process, the sample was pretreated. Dilute acid treatment was carried out by contacting the pulverized mass with 0.1 M HNO_3_ in a beaker with constant stirring for 1 h. Thereafter, diluted with excess distilled water and decanted, the dilution was repeated until the pH was 7.0. The residue was dried at 70 °C for 24 h in an oven, pulverized, and sieved through the 100-µm mesh. The prepared *Ananas comosus* peel waste (ACP) was kept in a desiccator until use.

One-pot synthesis by incipient-wet impregnation technique was applied in the preparation of the ZnO-biomass composite. The reducing action of the biogenic waste biomass in combination with chemical reduction was employed. This was carried out by adding 4.0 g of Zn(CH_3_COO)_2_ to 100 mL of distilled water in a beaker with vigorous stirring using a magnetic stirrer at 30 °C for 30 min. Thereafter, 10.0 g of pulverized *Ananas comosus* (without acid treatment) was added with continuous stirring for 15 min, after which 0.2 M NaOH solution was added dropwise until pH 11.0. The solution was stirred for 2 h, allowed to settle for 30 min, before centrifuging at 8000 rpm for 1 h. The obtained combo was washed repeatedly with distilled water until neutral pH and then centrifuged. The ZnONP biomass hybrid was dried at 80 °C for 24 h, pulverized using the mortar and pestle, sieved through a 100-µm screen, and designated as ZnO@ACP.

### Materials characterization

The ACP and ZnO@ACP were characterized to determine the surface properties. The morphology and elemental composition were analyzed by the field emission scanning electron microscopy (SEM; Jeol JSM-7800F model) coupled with energy-dispersive X-ray spectroscopy (EDS; Oxford X-max 80 mm^2^). The pore properties and surface area (S_BET_) were examined by the surface area analyzer (Micromeritics ASAP 2020 model) and results refined by MicroActive VI.01 software. The thermo-gravimetric analyzer (TGA; SDTA851e Mettler Toledo Model) with a 10 °C min^−1^ heating rate and 200 mL min^−1^ nitrogen flow rate analyzed the thermal stability. The pH drift method was used to determine the pH point of zero charged (pHpzc)^11^. The surface functionality was examined by the Fourier transform infrared spectrometer (FTIR; Brucker Model). X-ray diffraction (XRD) was determined using an X-ray diffractometer (Brucker Model) with Cu radiation of 1.54 Å. Diffraction patterns were in the 2θ range of 10–80°, with a step size of 0.1° and 2 s counting time per step.

### Adsorption of CEB

Exactly 0.1 g of CEB was dissolved in 1 L volumetric flask to obtain a stock solution of concentration 100 mg L^−1^. Serial dilution of the stock was employed to obtained concentrations of 10–50 mg L^−1^. The batch technique was used to evaluate the dependence of CEB adsorption, on contact time (10–180 min), pH (2.0–9.0), temperature (300–323 K), and CEB concentration (10–50 mg L^−1^). The solution pH was adjusted with 0.1 M solution of HCl or NaOH. In a typical experiment, 0.05 g of the material was added to 10 mL of a given CEB solution, agitated for 5 min and left for a contact time of 180 min. Thereafter, the solution was centrifuged at 8000 rpm for 30 min, analyzed for residual CEB concentration at the maximum wavelength of 644 nm (Supplementary Fig. S1, supplementary material), using the UV spectrophotometer (Shimadzu UV-1800 model). The studied parameter was varied while keeping the others constant. The adsorption of CEB was performed at pH 7.0, temperature 300 K, and dye concentration of 50 mg L^−1^. The adsorption capacity qe (mg g^−1^) and percentage removal were calculated from the mass balance and percentage equations, respectively^29^.

1 $$ qe \, = \, \left[ {\left( {C_{o} {-} \, C_{e} } \right)v} \right]/m $$

2 $$ R\left( \% \right) \, = \, \left[ {\left( {C_{o} {-} \, C_{e} } \right){/}C_{o} } \right]100 $$

where *v* (L) is the volume of solution*, m* (g) is the adsorbent weight, *C*_*o*_ and *C*_*e*_ in mg L^−1^ are the initial and residual CEB concentrations, respectively.

### Desorption and material reuse

The regeneration and reusability of the materials were evaluated by contacting 0.25 g of the adsorbent with 10 mL of 50 mg L^−1^ CEB solution at pH 7.0, a contact time of 180 min and temperature of 300 K. Thereafter, the solution was centrifuged and the amount of CEB remaining was determined by the UV spectrophotometer. The CEB-loaded material was oven-dried at 70 °C for 30 min, after which it was mixed with 10 mL of 0.2 M HCl eluent, agitated for 5 min, and left for a contact time of 25 min at 300 K. The concentration of CEB in the desorbed acidic solution was analyzed after centrifugation. The desorption of CEB was evaluated from equation^30^:

3 $$ \% \, Desorption \, = \, 100\left[ {C_{D} V_{D} } \right]/q_{e} m $$

where V_D_ (L) is the volume of eluent used and C_D_ (mg L^−1^) represents the concentration of CEB in the acidic desorbed solution. The parameter, q_e_ (mg g^−1^) denotes the adsorption capacity of the material for CEB, m (g) represents the adsorbent’s mass. The regenerated adsorbents after oven drying at 80 °C for 30 min, was reused for CEB adsorption. Three cycles of adsorption–desorption experiments were carried out.

### Statistics

The best fit kinetic or isotherm model describing adequately the adsorption data was obtained from the coefficient of determination (R^2^), calculated from the statistical function of Origin 2019b software. The closer the R^2^ value to one the best the model fit. The experimental responses were evaluated using descriptive statistics. Each experimental run was carried out in triplicate and the mean values computed. The figures were plotted using the origin 2019b software and the error bars in the figures represent the standard deviations where applicable.

## Results and discussion

### Characterization of ACP and ZnO@ACP

**Figure 1:**
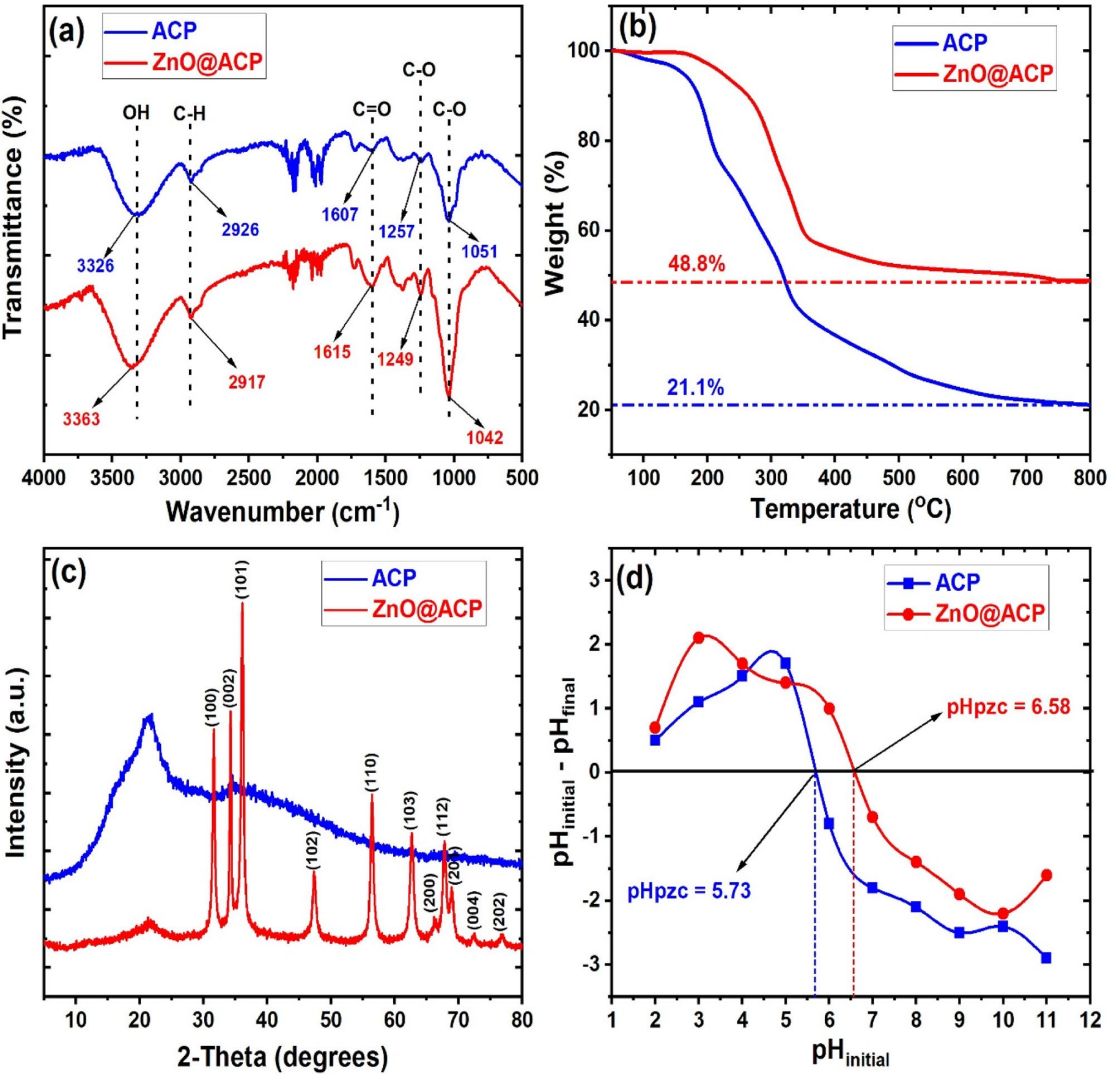
The (**a**) Fourier transform infrared; (**b**) thermogravimetric analysis; (**c**) X-ray diffraction; (**d**) pH point of zero charge of ACP and ZnO@ACP.

**Figure 2:**
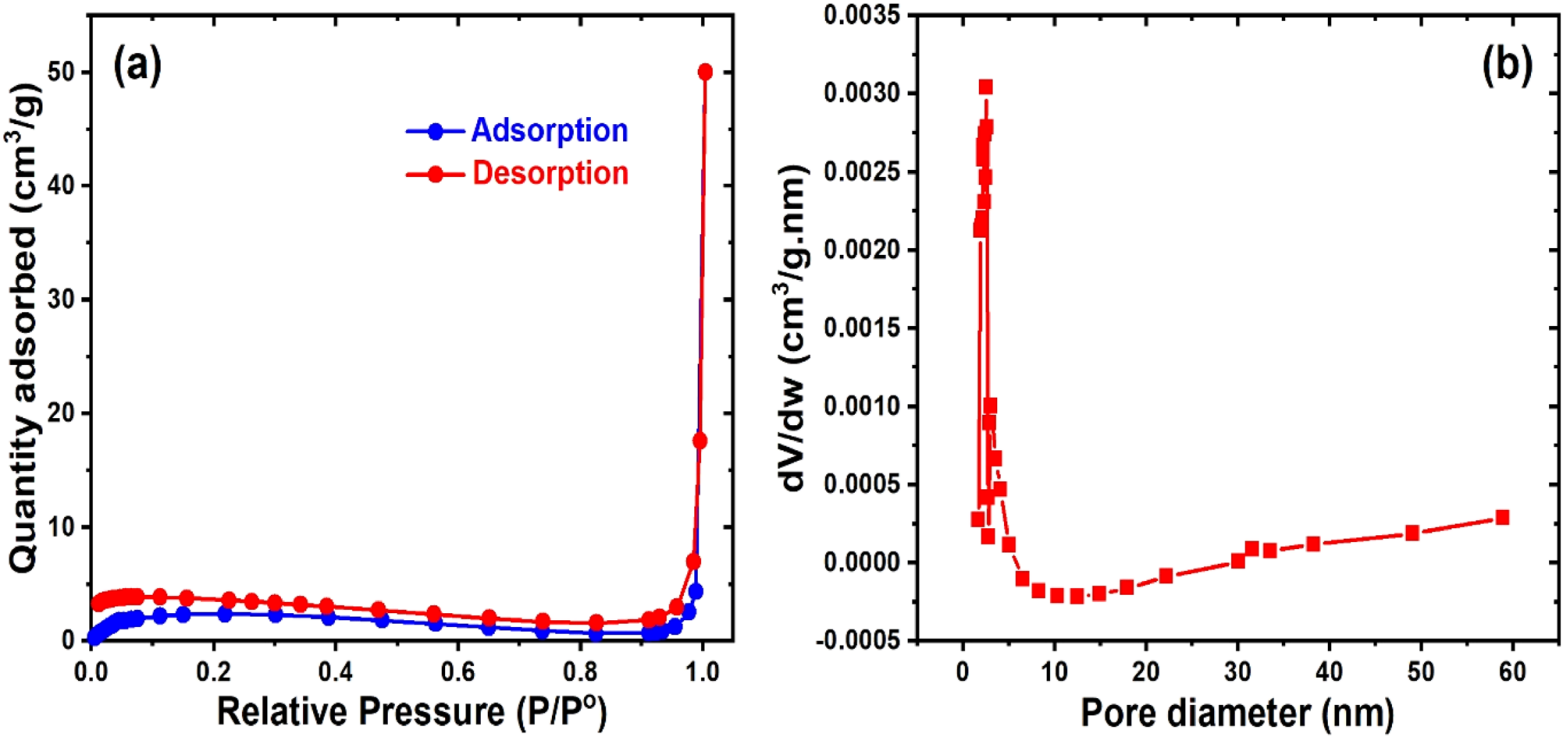
The (**a**) nitrogen adsorption–desorption isotherm at 77 K and (**b**) pore analysis of ZnOACP.

**Figure 3:**
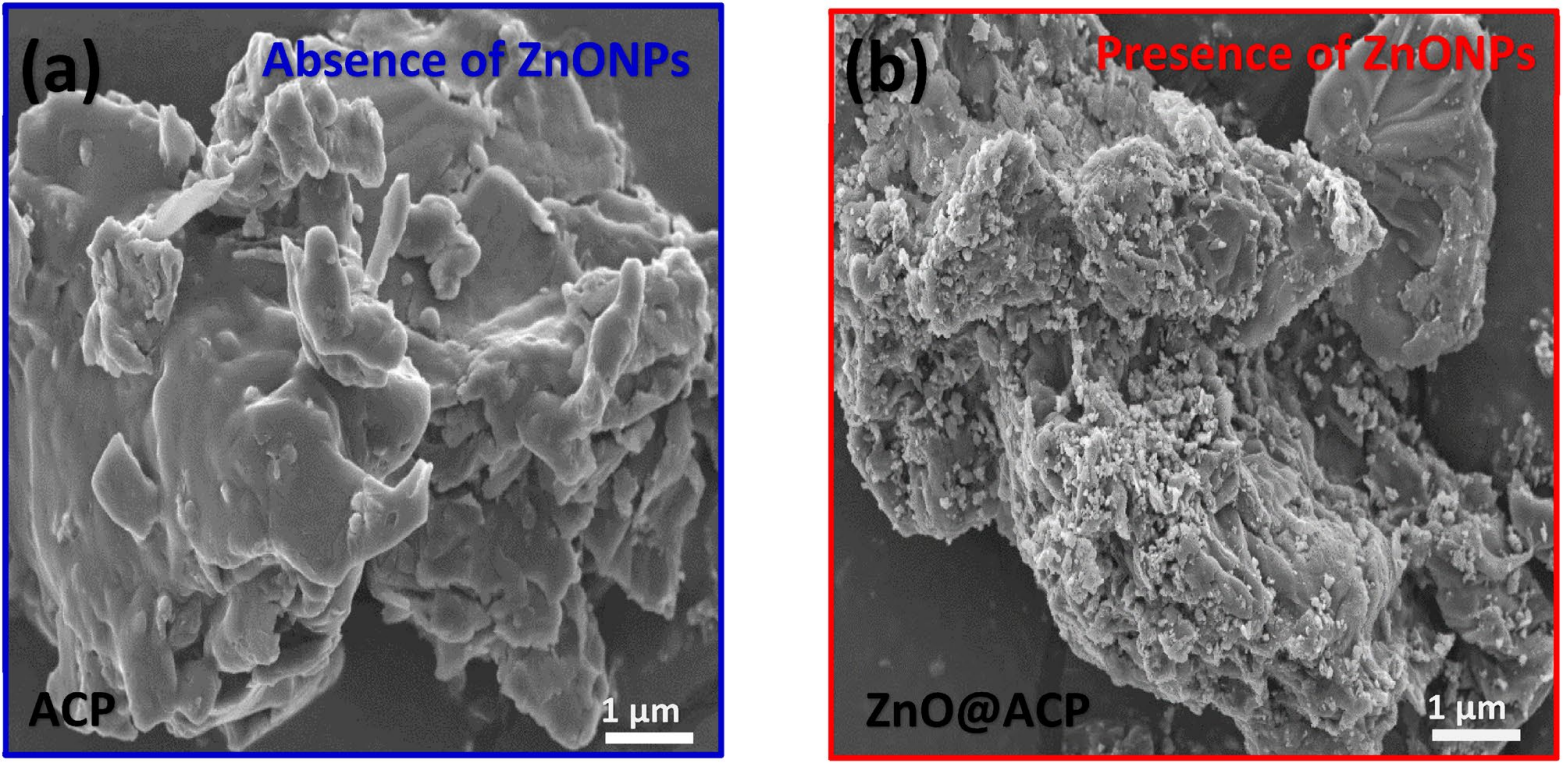
The Scanning electron microscopy of (**a**) ACP and (**b**) ZnO@ACP.

The characterization of ACP and ZnO@ACP is shown in Figs. 1, 2 and 3. The surface functional groups on the materials presented by the Fourier transform infrared spectra is illustrated in Fig. 1a. The presence of functional groups such as OH, C=O, and C‒O was observed. Shifts in absorption bands of these functional groups after ZnONPs impregnation shows the fixing of the metallic NPs on these sites. The occurrence of significant surface functionality on the materials implies efficient potentials for CEB uptake from solution^10^. The thermo-gravimetric analysis (TGA) of the adsorbents (Fig. 1b) showed higher thermal stability of ZnO@ACP compared to ACP. At 800 °C, ZnO@ACP retain almost half the initial weight of the material. This indicated that ZnONP loading enhanced the thermal stability of the biomass. The X-ray diffraction (XRD) used to examine the crystal phases and crystallinity of the materials is shown in Fig. 1c. The ACP presented a diffraction pattern typical of biomaterials. The occurrence of several well-defined new diffractions for ZnO@ACP, at 31.6°, 34.2°, 36.1°, 47.3°, 56.4°, 62.7°, 66.1°, 67.9°, 69.1°, 73.4°, and 77.3°, corresponding to the hexagonal phase of ZnO lattice planes, clearly confirms successful incorporation of ZnONPs on ACP. These diffractions are well-matched with the standard JCPDS card no. 36-1451^31^. The average crystalline size of ZnONPs calculated from the Debye–Scherrer’s equation using the main characteristic peak at 26.1° was 31.4 nm. The presence of ZnONPs in the hybrid increased the pHpzc of the biomass (Fig. 1d), which implies optimum uptake of the cationic CEB is likely to be favored at higher pH values than the pHpzc. From the nitrogen-adsorption/desorption isotherm and porosity analysis, the Brunauer–Emmett–Teller surface area (S_BET_), pore volume, and pore diameter of ACP was 39.816 m^2^ g^−1^, 0.0245 cm^3^ g^−1^, and 1.184 nm, respectively. As shown in Fig. 2, ZnO@ACP exhibited a Type III isotherm according to the IUPAC classification with S_BET_ of 10.564 m^2^ g^−1^, a pore-volume of 0.0068 m^2^ g^−1^, and pore diameter of 1.761 nm. Despite the decrease in S_BET_ after the impregnation of ZnONPs, the increase in pore diameter could be beneficial for efficient dye uptake^32^. The scanning electron microscopy (SEM) of ACP (Fig. 3a) and ZnO@ACP (Fig. 3b) clearly shows the presence of impregnated ZnONPs on the surface of the latter, which was absent on ACP. This was supported by the energy-dispersive X-ray (EDX) results (Supplementary Fig. S2, supplementary material), which shows the occurrence of Zn in ZnO@ACP, but absent in ACP. The ZnONPs were agglomerated on the surface of ACP in the hybrid adsorbent with an average size of 35.7 nm (Supplementary Fig. S3, supplementary material). These characterizations indicate the successful synthesis of the ZnO-biomass nanocomposite.

### Influencing parameters

**Figure 4:**
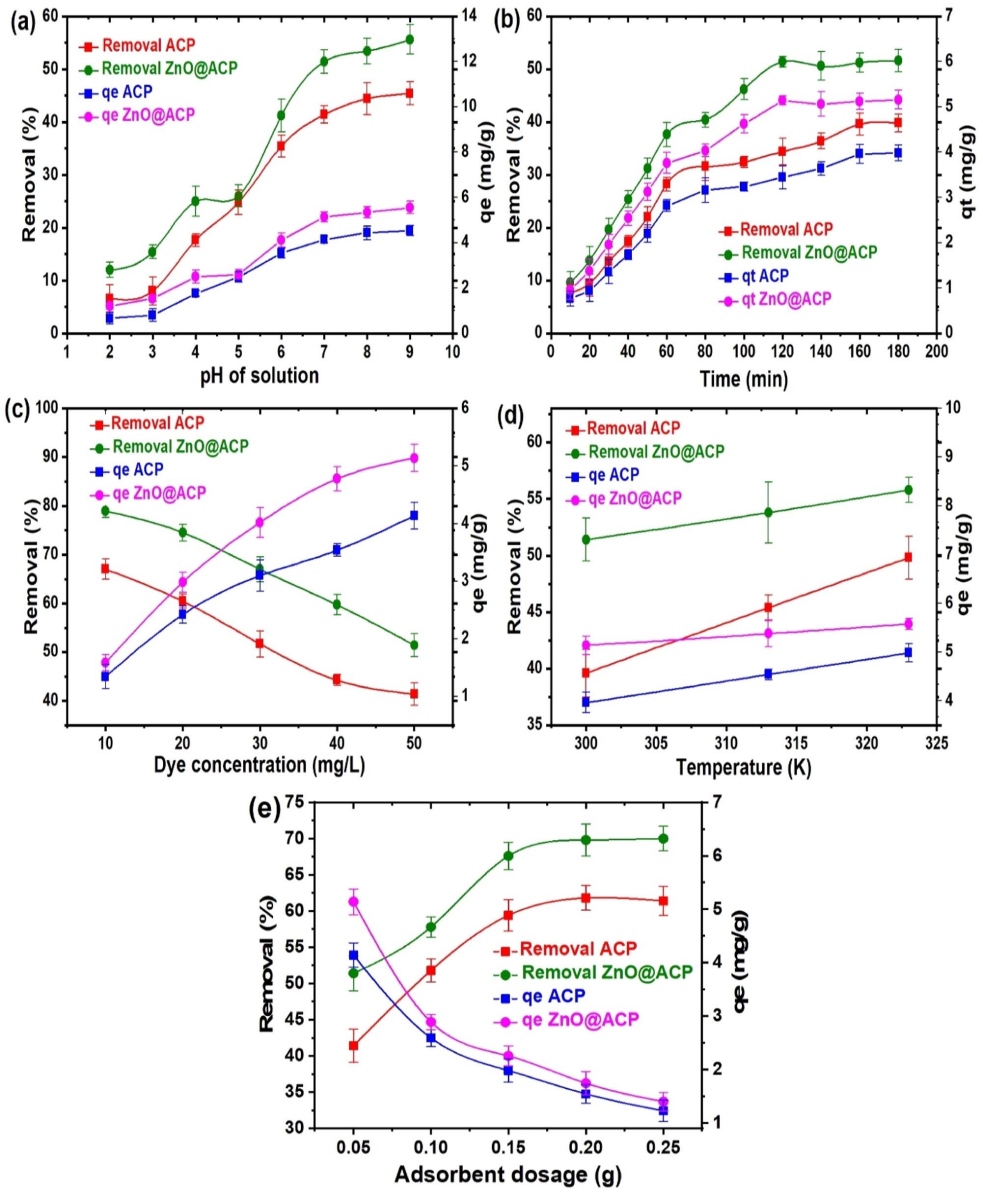
Effect of (**a**) solution pH (C_o_ = 50 mg L^−1^, dosage = 0.05 g, T = 300 K, t = 180 min) (**b**) contact time (pH = 7.0, dosage = 0.05 g, C_o_ = 50 mg L^−1^, T = 300 K), (**c**) dye concentration (pH = 7.0, dosage = 0.05 g, T = 300 K, t = 180 min), (**d**) temperature (pH = 7.0, dosage = 0.05 g, t = 180 min, C_o_ = 50 mg L^−1^) and (**e**) adsorbent dosage (pH = 7.0, T = 300 K, t = 180 min, C_o_ = 50 mg L^−1^) on the adsorption of celestine blue onto ACP and ZnO@ACP.

Several factors such as solution temperature, pH, time, and dye concentration are known to influence significantly the uptake of dyes on adsorbents^33^. The effect of solution pH on the uptake of CEB onto ACP and ZnO@ACP is shown in Fig. 4a. We observed an increase in CEB adsorption with an increase in pH. As predicted, based on the pHpzc, optimum CEB uptake occurred at pH values higher than the pHpzc of the materials. We maintained a pH of 7.0 in the adsorption to avoid cationic dye precipitation associated with higher pH values^9^. ZnO@ACP exhibited a faster equilibrium uptake of 120 min for CEB when compared to 160 min for ACP when the contact time was varied (Fig. 4b). This implies that ZnONPs impregnation increased the rate of adsorption. This faster rate is significant in reactor design application for the practical treatment of industrial effluent. The abundant adsorption sites on the materials accounted for the fast CEB uptake at the initial stages^34^. These sites were saturated as time progresses leading to the equilibrium CEB uptake. The influence of CEB concentration on its uptake onto ACP and ZnO@ACP is presented in Fig. 4c. The trend of increase in adsorption capacity and a decrease in percentage removal of CEB with an increase in dye concentration was observed. The increase in adsorption capacity of the materials with CEB concentration is due to higher dye concentration enhancing better interaction and subsequent fixing on the active sites^35^. On the other hand, the decrease in percentage uptake is attributed to the saturation of the sites on ACP and ZnO@ACP at higher CEB concentration^36^. A similar pattern was obtained in the adsorption of cationic methylene blue dye onto mango leaf powder^37^. Also, the higher temperature was found to favor the uptake of CEB on the materials (Fig. 4d), which suggested an endothermic CEB uptake^9^. The effect of material dosage on CEB adsorption is shown in Fig. 4e. As observed, with increasing dosage from 0.05 to 0.25 g, an increase in percentage uptake from 41.4 to 61.5% and from 51.6 to 70.2% was obtained for ACP and ZnO@ACP, respectively. This is due to the presence of more adsorption sites for CEB uptake with increasing dosage^38^. However, there was no significant change in CEB adsorption on both adsorbents with a further increase in dosage from 0.15 to 0.25 g, which is attributed to aggregation of adsorption sites with excess dosage^39^. On the other hand, we noticed a significant decrease in the adsorption capacity of the materials with an increase in dosage. This decrease has been associated with increasing adsorption sites resulting in less utilization due to aggregation of the sites as stated earlier. Hence, 0.05 g of the materials were selected for CEB adsorption, since maximum use of the adsorbent sites was exploited for CEB uptake. These trends obtained for CEB adsorption on the materials are similar to those reported by many researchers for cationic dyes^7,40–43^. Interestingly, ZnO@ACP recorded higher adsorption than ACP under variations of the experimental factors, which showed the efficacy of ZnONP in enhancing the uptake of the biomass for CEB.

### Adsorption mechanism and materials regeneration

**Figure 5:**
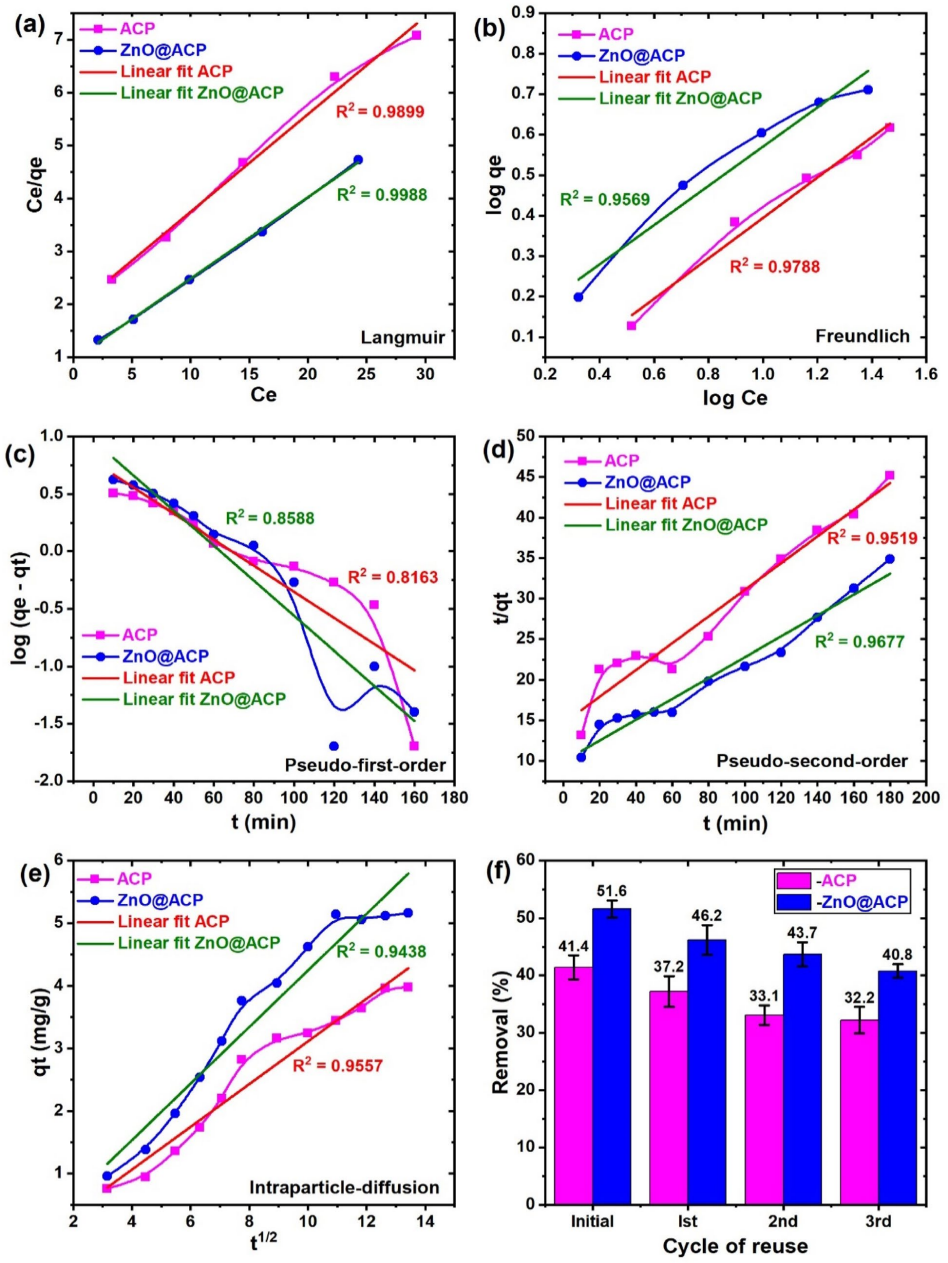
The (**a**–**e**) adsorption isotherm and kinetic modeling and (**f**) reusability of the materials for celestine blue adsorption.

The mechanism of CEB uptake onto ACP and ZnO@ACP was evaluated from the kinetics, isotherm, and thermodynamic. The models’ descriptions are presented in the supplementary material. The isotherm of CEB adsorption on the materials was evaluated by Freundlich and Langmuir models and the plots are shown in Fig. 5a,b. The Langmuir isotherm was more suitable in the adsorption of CEB on both materials than the Freundlich isotherm indicating a monolayer uptake of CEB on homogenous material surfaces. This implies that the impregnated ZnONPs were uniformly distributed on the hybrid. Table 1 shows that the maximum monolayer uptake of CEB on ACP (5.42 mg g^−1^) was slightly improved with ZnO@ACP (6.52 mg g^−1^). The Langmuir R_L_ value of 0.171–0.508 for ACP and 0.110–0.383 for ZnO@ACP as well as the Freundlich n values indicated an efficient affinity for CEB^44^. The maximum adsorption capacity of ACP and ZnO@ACP for CEB was compared with other low costs, pineapple, and ZnONPs based adsorbents used for cationic dye adsorption as shown in Table 2. Although the uptake of CEB on the as-prepared adsorbents was higher than some low-cost adsorbents for cationic dyes, it was quite low compared to the pineapple and ZnONP based adsorbents. This suggests a low uptake of CEB relative to other cationic dyes. However, future researches on the adsorption of CEB on materials is needed to enable proper evaluation and to arrive at a more valid conclusion.

**Table 1:** The isotherm, kinetic and thermodynamics for the adsorption of celestine blue.

Model	Parameter	ACP	ZnO@ACP
Langmuir	K_L_ (L mg^−1^)	0.097	0.161
	q_L_ (mg g^−1^)	5.42	6.52
	R^2^	0.9899	0.9988
Freundlich	N	2.004	2.064
	K_F_ (L g^−1^)	0.79	1.22
	R^2^	0.9788	0.9569
Pseudo-first-order	K_I_ (min^−1^)	0.0262	0.0350
	qe_cal_ (mg g^−1^)	6.09	9.26
	R^2^	0.8163	0.8588
Pseudo-second-order	K_2_ (g mg^−1^ min)	1.86 × 10^–3^	1.66 × 10^–3^
	qe_cal_ (mg g^−1^)	6.07	7.78
	R^2^	0.9519	0.9677
Intraparticle diffusion	K_d_ (mg g^−1^ min^−1/2^)	0.341	0.452
	C	-0.299	-0.272
	R^2^	0.9557	0.9438
Thermodynamics	(300 K) ∆G° (kJ mol^−1^)	1.05	-0.139
	(313 K) ∆G° (kJ mol^−1^)	0.480	-0.398
	(323 K) ∆G° (kJ mol^−1^)	0.021	-0.626
	∆H° (kJ mol^−1^)	14.49	6.17
	∆S° (J molK^−1^)	44.79	21.03

**Table 2:** Comparison of the maximum adsorption capacity of the as-prepared adsorbents for celestine blue with other adsorbents used in the adsorption of cationic dyes.

Adsorbent	Cationic dye	qe (mg g^−1^)	References
Pineapple peel	Crystal violet	158.73	^45^
Pineapple peel	Methylene blue	97.09	^46^
Pineapple leave	Crystal violet	72.39–78.23	^47^
Modified pineapple peel	Methylene blue	52.6	^48^
Sugarcane bagasse	Rhodamine B	44–51.3	^49^
Pineapple leaf activated carbon	Methyl violet	31.24	^50^
Sugarcane bagasse	Basic blue 9	25.3–28.0	^49^
Seaweed-ZnO-polyaniline composite	Methylene blue	20.55	^51^
Magnetic pineapple leaf activated carbon	Methyl violet	16.76	^50^
ZnO-chitosan composite	Malachite green	11.0	^52^
ZnO hybrid beads	Basic blue 41	1.0–8.0	^53^
ZnO@ACP	Celestine blue	6.52	This study
ACP	Celestine blue	5.42	This study
Neem sawdust	Crystal violet	4.44	^54^
Poultry feathers	Malachite green	3.55	^52^
Brewery spent grain	Malachite green	2.55	^52^
Polylactide/spent grain	Malachite green	1.48	^52^
ZnO nanoparticle	Methylene blue	0.3428	^55^

The kinetics of CEB uptake was evaluated by the pseudo-first order and pseudo-second-order rate equations, while the diffusion mechanism by the intraparticle diffusion model. The kinetic model plots are shown in Fig. 5c–e. It is obvious from the plots that the pseudo-first-order model was more appropriate at the initial stages of adsorption up to the equilibrium time after which the pseudo-second order was more fitted. However, based on the R^2^ values, the pseudo-second-order kinetics presented the best fit in the overall adsorption of CEB on both ACP and ZnO@ACP than the pseudo-first-order model. This suggests the involvement of electrostatic interactions between CEB molecules in solution and the surfaces of ACP and ZnO@ACP^56^. The involvement of the intraparticle-diffusion mechanism was found to be the dominant diffusion mechanism for CEB uptake. However, we observed the presence of intercept values, showing that the plot did not pass through the origin. The implication is that boundary layer diffusion was also involved in the overall removal process of CEB on the materials and that the uptake was not restricted solely to the intraparticle diffusion mechanism^39^. The thermodynamic analysis showed that the uptake of CEB on ACP is non-spontaneous based on positive ∆G^o^ values. Therefore, the initial agitation and high CEB concentration created a driving force for efficient interaction between CEB molecules and ACP. However, after the impregnation of ZnONPs on ACP, the uptake became spontaneous as indicated by negative Gibbs free energy changes at all temperatures, which is desirable (Table 1). An endothermic uptake of CEB on both materials was deduced based on positive ∆H^o^ values, which supported the increase in CEB adsorption with temperature observed in Fig. 4d. Physical adsorption of CEB on both materials was suggested, ascribed to the ∆H^o^ values in the range of 2.1–20.9 kJ mol^−1^^10^. Similar results were reported for other cationic dyes^9,57^.

**Figure 6:**
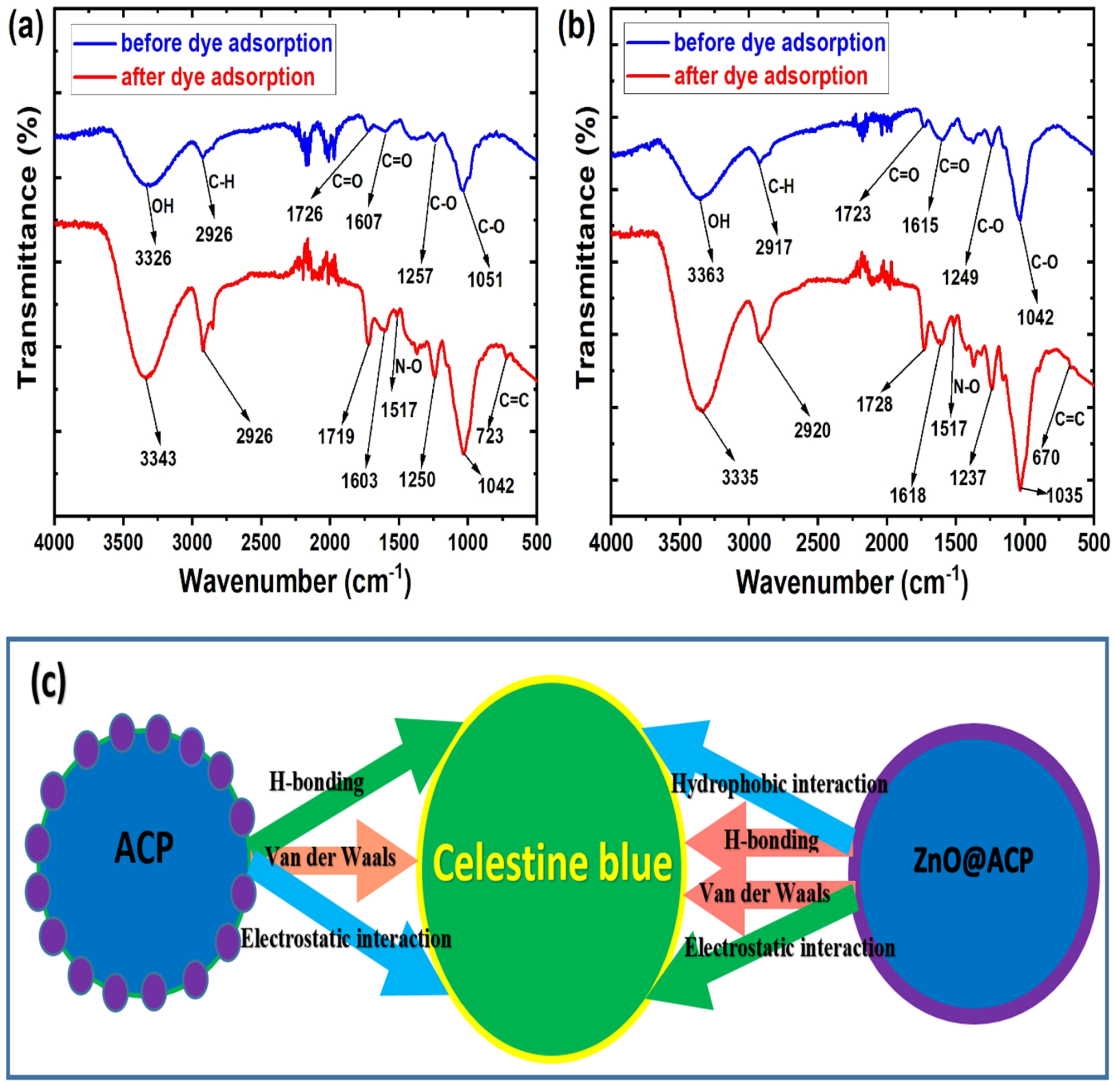
The FTIR spectra of (**a**) ACP and (**b**) ZnO@ACP before and after the adsorption of celestine blue, and (**c**) the schematic representation of the deduced adsorption mechanism.

The mechanism of CEB uptake on ACP and ZnO@ACP was evaluated from the FTIR spectra before and after adsorption, to ascertain the functional sites used in the removal process (Fig. 6). For ACP, band shifts in the OH, C=O, and C–O functionalities were observed, with the occurrence of new bands corresponding to the N–O and C=C groups of CEB. Besides, the absorptions were more intense due to the presence of CEB molecules, while there was no shift in the C–H absorption band. These observations prove that the mechanism of CEB adsorption on ACP is due to the H-bonding, Van der Waals, and electrostatic interactions^32^. A similar deduction applies to the ZnO@ACP composite, except for the shift in the C–H bands suggesting additional hydrophobic interaction. The schematic representation of the mechanism of CEB uptake onto ACP and ZnO@ACP is shown in Fig. 6c. Furthermore, a viable material applied for pollutant removal apart from high uptake must be associated with good regeneration and reusability^58^. This is to avoid the accumulation of excess pollutant-loaded materials in the environment after the treatment process. Thus, the regeneration and reuse of ACP and ZnO@ACP for CEB adsorption was evaluated as shown in Fig. 5f. High CEB desorption of 77.5% (ACP) and 81.3% (ZnO@ACP) from the dye-loaded material was achieved using 0.2 M HCl solution. This shows the efficient potentials, most especially for the composite material to be regenerated. Although there was a slight decrease in CEB adsorption after each successive cycle of reuse, the efficacy of the materials to be regenerated and reused for CEB adsorption was established. ZnO@ACP also displayed higher CEB uptake than the pristine ACP at the initial as well as the cycles of reuse, which shows the efficacy of ZnONPs in enhancing the biosorption of CEB from solution.

### Conclusions

The impregnation of ZnONPs onto biowaste (ACP) to form a hybrid (ZnO@ACP) was found to enhance the adsorption of celestine blue (CEB) at variations of pH, temperature, time, adsorbent dosage, and dye concentration. The SEM, EDX, and XRD analysis showed successful impregnation of 35.7 nm-sized ZnONPs on the hybrid. The presence of ZnONPS increased the thermal stability as well as the pH point of zero charge of the biosorbent. The dye uptake on both adsorbents was well explained by the Langmuir isotherm and pseudo-second-order kinetic models. The intraparticle diffusion mechanism was found to be the major mechanism of dye removal, while thermodynamics revealed that the presence of ZnONPS enhanced the spontaneity for CEB adsorption. We found that ZnO@ACP exhibited a faster uptake of CEB when compared to the pristine ACP. The hybrid also exhibited higher dye desorption from its loaded surface as well as higher adsorption over three cycles of regeneration and reuse. The adsorption behavior of CEB was similar to other cationic dyes reported in the literature. These results showed the viability of ZnO@ACP for CEB decontamination from wastewater. In addition, we recommend that more studies should be conducted on the adsorption of CEB on various materials to provide sufficient insights into the adsorption behavior.

## Supplementary information

Supplementary file1.
