# Supplementary material for: Synthesis, characterization, and regeneration of an inorganic–organic nanocomposite (ZnO@biomass) and its application in the capture of cationic dye
Source: Sci Rep. 2020 Sep 2;10:14441. doi: 10.1038/s41598-020-71261-x (PMC7468233; doi:10.1038/s41598-020-71261-x)
Supplement: Supplementary file 1 — Supplementary file1. [file 41598_2020_71261_MOESM1_ESM.docx]

**Synthesis, characterization, and regeneration of an inorganic-organic nanocomposite (ZnO@biomass) and its application in the capture of cationic dye**

**Kovo G. Akpomie^1,2*^, Jeanet Conradie^1^**

^1^Department of Chemistry, University of the Free State, Bloemfontein, South Africa

^2^Department of Pure & Industrial Chemistry, University of Nigeria, Nsukka.

*[kovo.akpomie@unn.edu.ng](mailto:kovo.akpomie@unn.edu.ng); [kovoakpmusic@yahoo.com](mailto:kovoakpmusic@yahoo.com)

**Supplementary Material**

**Adsorption isotherms**

The Freundlich and Langmuir isotherm models were utilize to evaluate the affinity of the As-prepared materials for CEB ^1^. The Freundlich isotherm corresponds to multilayer uptake onto heterogeneous material surfaces and is represented as:

*log q_e_ = log K_F_ + [1/n]log C_e_* (4)

Where *n* and *K_F_* (L/g) corresponds to the Freundlich adsorption intensity and capacity, respectively. Efficient affinity between the adsorbate and adsorbent exist if n values lie between 1 and 10. On the other hand, the Langmuir isotherm depicts a monolayer uptake of the adsorbate onto homogenous surface of the materials, and is expressed as:

*C_e_/q_e_ = 1/q_L_K_L_ + C_e_/q_L_*  (5)

Where *q_L_* (mg/g) corresponds to the maximum monolayer adsorption and *K_L_* (L/mg) is the Langmuir constant. A Langmuir dimensionless parameter (R_L_), provides insight into the nature of adsorption and is represented as:

R_L_ = 1/[1 + K_L_C_o_] (6)

Values of R_L_ depicts favorable removal (1 > R_L_ > 0), unfavorable removal (R_L_ > 1), linear uptake (R_L_ =1) and irreversible removal (R_L_ = 0).

**Adsorption Kinetics**

The CEB adsorption kinetics onto the materials was evaluated by the pseudo-first-order (PF), pseudo second order (PS) and intraparticle diffusion (ID) rate equation ^2,3^. The PF equation is expressed linearly as:

*log(q_e_ – q_t_) = log q_e_ – (K_I_t/2.303)*  (7)

Where *t* (min) is the contact time of removal, *q_t_* (mg/g) is the adsorption capacity at a given time *t* (min) and *K_I_* (min^-1^) is the PF rate constant. The PS equation is expressed linearly as:

*t/q_t_ = 1/(K_2_qe^2^) + t/q_e_* (8)

Where *K_2_* (g/mg min) represents the PS rate equation. The ID used to provide information on diffusion mechanism is expressed as:

*q_t_ = K_d_t^1/2^ + C*  (9)

Where *K_d_* (mg/g min^1/2)^ and C represents the ID rate constants and intercept, respectively. The mechanism of adsorption is intra-particle diffusion based if a linear plot of *q_t_* versus *t^1/2^* is obtained; otherwise, it is surface diffusion dependent.

**Thermodynamic analysis**

Thermodynamics of CEB adsorption on the adsorbents to evaluate the enthalpy change (ΔH^o^), entropy change (ΔS^o^) and Gibb’s free energy change (ΔG^o^) were carried out by the application of the following equations ^4^:

*ΔG^o^ = ̶ RTln K_c_*  (10)

*ln K_c_ = ̶ (ΔH^o^/RT) + (ΔS^o^/R)* (11)

Where T (K), *R* (J mol^-1^ K^-1^) and *K_c_* corresponds to the absolute temperature, ideal gas constant, and distribution coefficient, respectively.

**Figures**

**
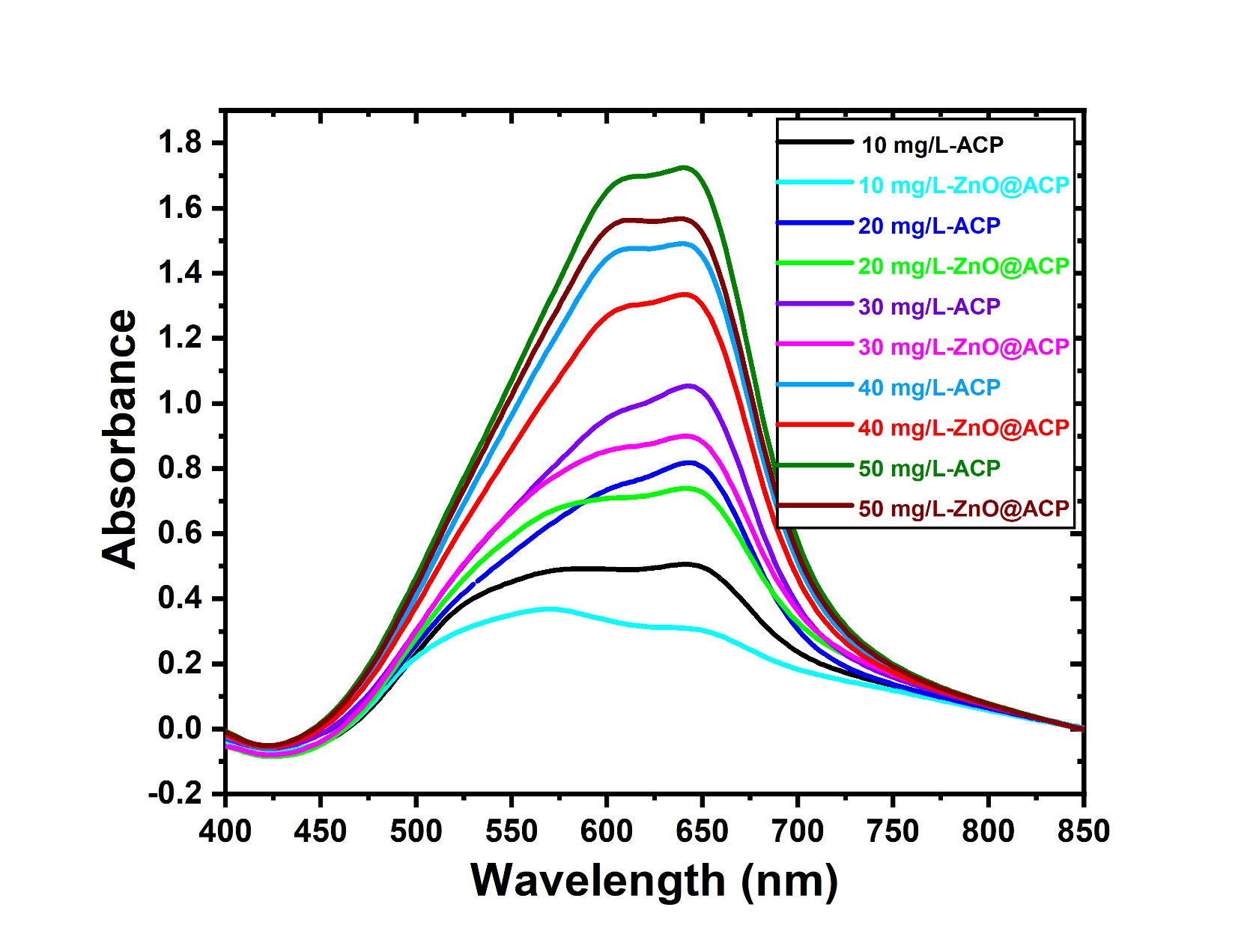
**

**Fig. S1:** The UV spectra for the effect of initial dye concentration after dye adsorption, showing the maximum Celestine blue absorbance at 644 nm.


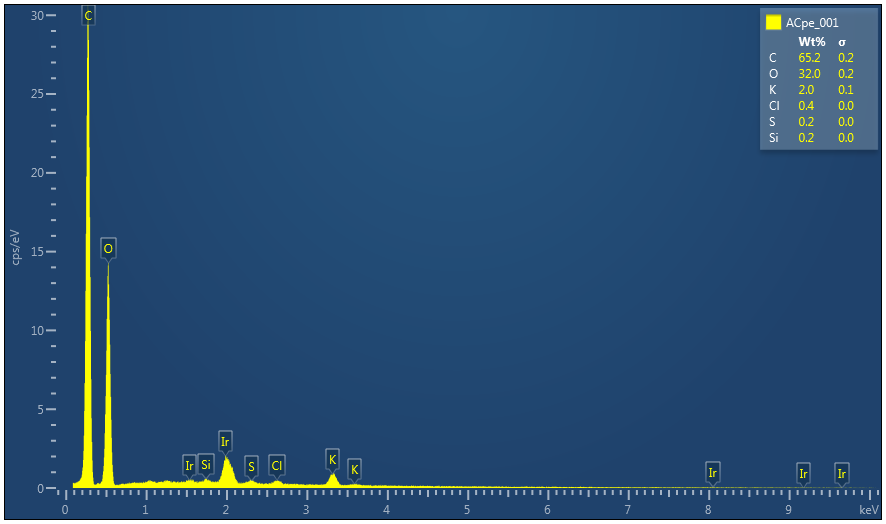


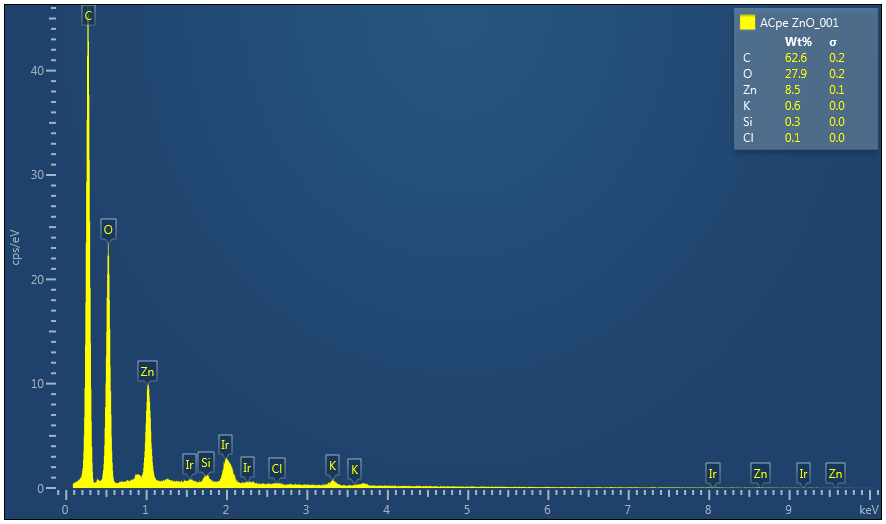


**Fig. S2: Energy-dispersive X-ray (EDX) analysis of ACP and ZnO@ACP**





**100 nm**

**ZnONPs**

**
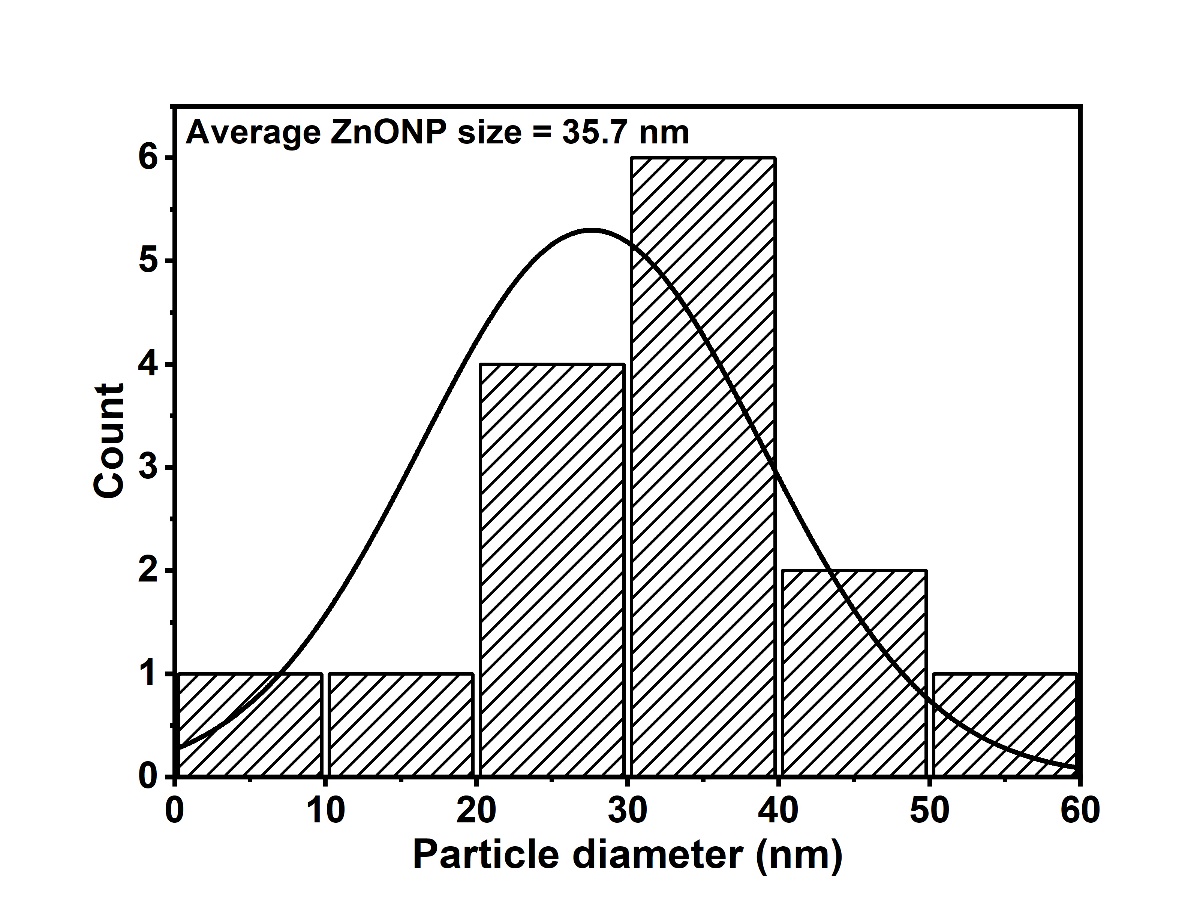
**

**Fig. S3: The Scanning electron microscopy of the ZnONPs impregnated on ACP and the average particle size.**

**References**

1. Xu, B. *et al.* Electrospinning preparation of PAN/TiO2/PANI hybrid fiber membrane with highly selective adsorption and photocatalytic regeneration properties. *Chem. Eng. J.* **399**, 125749 (2020).

2. Shin, J. *et al.* Single and competitive adsorptions of micropollutants using pristine and alkali-modified biochars from spent coffee grounds. *J. Hazard. Mater.* 123102 (2020) doi:10.1016/j.jhazmat.2020.123102.

3. Chukwuemeka-Okorie, H. O., Ekemezie, P. N., Akpomie, K. G. & Olikagu, C. S. Calcined corncob-kaolinite Combo as new sorbent for sequestration of toxic metal ions from polluted aqua media and desorption. *Front. Chem.* **6**, 1–13 (2018).

4. Dawodu, M. O. & Akpomie, K. G. Evaluating the potential of a Nigerian soil as an adsorbent for tartrazine dye: Isotherm, kinetic and thermodynamic studies. *Alexandria Eng. J.* **55**, 3211–3218 (2016).
